# The varying extent of humoral and cellular immune responses to either vector- or RNA-based SARS-CoV-2 vaccines persists for at least 18 months and is independent of infection

**DOI:** 10.1128/jvi.01912-23

**Published:** 2024-03-19

**Authors:** Franz Mai, Wendy Bergmann, Emil C. Reisinger, Brigitte Müller-Hilke

**Affiliations:** 1 Core Facility for Cell Sorting and Cell Analysis, University Medical Center, Rostock, Germany; 2 Division of Tropical Medicine and Infectious Diseases, Center of Internal Medicine II, University Medical Center, Rostock, Germany; 3 Institute of Immunology, University Medical Center, Rostock, Germany; University of North Carolina at Chapel Hill, Chapel Hill, North Carolina, USA

**Keywords:** SARS-CoV-2, COVID-19, homologous and heterologous vaccination regime, T cell memory, antibodies

## Abstract

The corona virus disease 2019 (COVID-19) pandemic caused by severe acute respiratory syndrome corona-virus 2 (SARS-CoV-2) spurred a worldwide race for the development of an efficient vaccine. Various strategies were pursued; however, the first vaccines to be licensed presented the SARS-CoV-2 spike protein either in the context of a non-replicating adenoviral vector or as an mRNA construct. While short-term efficacies have extensively been characterized, the duration of protection, the need for repeated boosting, and reasonable vaccination intervals have yet to be defined. We here describe the adaptive immune response resulting from homologous and heterologous vaccination regimen at 18 months after primary vaccination. To that extent, we monitored 176 healthcare workers, the majority of whom had recovered from previous SARS-CoV-2 infection. In summary, we find that differences depending on primary immunization continue to exist 18 months after the first vaccination and these findings hold true irrespective of previous infection with the virus. Homologous primary immunization with BNT162b2 was repeatedly shown to produce higher antibody levels and slower antibody decline, leading to more effective *in vitro* neutralization capacities. Likewise, cellular responses resulting from *in vitro* re-stimulation were more pronounced after primary immunization involving BNT162b2. In contrast, IL-2 producing memory T helper and cytotoxic T cells appeared independent from the primary vaccination regimen. Despite these differences, comparable infection rates among all vaccination groups suggest comparable real-life protection.

**IMPORTANCE:**

Vaccination against the severe acute respiratory syndrome corona-virus 2 (SARS-CoV-2) was shown to avert severe courses of corona virus disease 2019 (COVID-19) and to mitigate spreading of the virus. However, the duration of protection and need for repeated boosting have yet to be defined. Monitoring and comparing the immune responses resulting from various vaccine strategies are therefore important to fill knowledge gaps and prepare for future pandemics.

## INTRODUCTION

The emergence of the novel severe acute respiratory syndrome corona virus 2 (SARS-CoV-2) in December 2019 caused a pandemic that led not only to millions of deaths worldwide but also to social and economic restrictions of an unprecedented extent. Also unprecedented was the global race for new vaccines. At warp speed, established and novel strategies were pursued in order to deploy the spike protein and elicit protective immune responses (1, 2). Indeed, the first vaccines that were validated in clinical studies and licensed were based on mRNA constructs and non-replicating adenoviral vectors, respectively (3–5). Nationwide immunization campaigns started in Germany in December 2020, and even though they turned out to not fully prevent infections, severe courses of corona virus disease 2019 (COVID-19) could be diminished (6, 7). Likewise, the risk of developing long COVID—the sustained sequelae following infection with SARS-CoV-2—seems to be reduced after vaccination (8).

The magnitude of individuals being vaccinated worldwide and within a relatively short timeframe allowed for an in-depth monitoring of the extent of B and T cell responses, the durability of neutralizing antibodies and spike-specific T cells, and differences between the various vaccines and vaccine regimen. It was thus shown that single-dose vaccination with either BNT162b2 or AZD1222 induced strong but disparate humoral and cellular immune responses. In detail, both vaccines elicited spike protein-specific effector T cells, while BNT162b2 causes the highest serum IgG antibody level (9–11). We ourselves have also shown that AZD1222 induced transient leukopenia, significant changes among innate and adaptive immune cell subpopulations, and a significant reduction of anti-inflammatory T cells after re-stimulation (9). Only few early time point studies exist showing that a single dose of BNT162b2 provided higher IgA and/or IgM than AZD1222 (9, 11, 12).

After two vaccine doses and 6 months after primary immunization, we and others were able to demonstrate that a heterologous prime boost regimen combining AZD1222 with any of the mRNA-based vaccines not only elicited a significantly higher number of antibodies but also improved neutralizing capacity (13–15). T cell responses were conspicuously comparable between the various vaccination regimen using AZD1222 and/or BNT162b2 (10, 13, 15). After three vaccine doses and 12 months after primary immunization, differences in antibody quantity resulting from the primary vaccination regimen were hardly discernible; however, primary vaccination including BNT162b2 impacted positively on the recognition of the omicron variant of concern (15–18). We here set out to analyze the spike-specific immune responses at 18 months after primary immunization. Increases in global infection rates allowed for the evaluation of direct virus contacts in addition to vaccination.

## MATERIALS AND METHODS

### Study participants and blood sampling

One hundred seventy-six employees from science, nursing, medical, technical, and functional services as well as from the administration of the University Medical Center Rostock were recruited via the local Coordination Center for Clinical Studies. No personal data except for age, sex, numbers of vaccinations, numbers, and approximate dates of infections and results from SARS-CoV-2-specific PCR tests were requested. Study participants were exclusively Caucasians except for two individuals of Asian and Syrian origin. EDTA and serum blood were obtained by venipuncture. Plasma and serum were processed via centrifugation at 1,500 *g* for 10 minutes and 2000 *g* for 10 minutes, respectively. Both were subsequently frozen at −80°C for later use. Peripheral blood mononuclear cells (PBMCs) were isolated from anticoagulated blood by density gradient centrifugation using Ficoll-Paque PLUS according to the manufacturer’s instructions (Cytiva, Marlborough, MA, United States). They were then stored at −80°C in fetal calf serum (FCS, Thermo Fisher, Waltham, MA, USA) containing 10% dimethyl sulfoxide (Sigma-Aldrich, St Louis, MO, USA) until later use.

### Quantification of anti-SARS-CoV-2 receptor binding domain- (RBD) IgG and anti-nucleocapsid antibodies

Electrochemiluminescence immunoassays (ECLIA) (Elecsys Anti-SARS-CoV-2 S, Roche, Mannheim, Germany) were performed according to the manufacturer’s instructions. Frozen patient serum was thawed and incubated with biotinylated and ruthenylated recombinant spike receptor binding domain (RBD) or nucleocapsid antigens, followed by the addition of streptavidin-conjugated microparticles. Chemiluminescence emitted from microparticles bound to electrodes was quantified on Cobas E411 (Roche Diagnostics, Mannheim, Germany). Measured U/mL correlated strongly with the international WHO standard (*U* = 0.972 * BAU; Pearson *r* = 0.99996) and were therefore equalized to BAU/mL.

### Neutralizing capacity against Wuhan-Hu-1 and B.1.1.529/BA.1 (Omicron)

Neutralizing capacities were assessed via the SARS-CoV-2 Surrogate Virus Neutralization Test (sVNT) kit (GenScript, New Jersey, USA). Frozen plasma samples were thawed and centrifuged at 10,000 *g* for 5 minutes to remove precipitates before further use. Depending on the spike variant, the samples were diluted to varying degrees. For neutralization against Omicron, the samples were diluted 1:10 in Dilution Buffer, while the samples for Wu-Hu-1 were diluted 100- to 1,500-fold to remain within a measurable range for this assay. Horseradish-peroxidase (HRP) peptides, one being Wuhan-Hu-1 [SARS-CoV-2 Spike protein (RBD, Avi & His Tag)-HRP] and the other being Omicron (SARS-CoV-2 Spike protein RBD-HRP, Omicron Variant, His Tag) (both from GenScript, New Jersey, USA) were diluted 1:1,000. Diluted samples and diluted HRP peptides were then combined in a 1:2 ratio. After incubation at 37°C for 30 minutes, samples were transferred to ELISA capture plates and incubated again at 37°C for 15 minutes. Followed by four wash cycles, the substrate was added and incubated in the dark for an additional 15 minutes at room temperature. After stopping the substrate reaction, plates were measured photometrically at 450 nm using the InfiniteM200 (Tecan, Männeheim, Switzerland). Calculation of neutralizing capacity based on optical density was performed according to the manufacturer’s instructions: neutralizing capacity = (1 − ODSample/ODNeg.Ctrl) × 100%.

### Anti-human interferon gamma ELISpot

Frozen PBMCs were gently thawed and washed once in RPMI cell culture medium containing 1 mM pyruvate, 2 mM L-glutamine, 10 mM HEPES, 10% FCS, and 100 U penicillin/0.1 mg streptomycin (PAN-Biotech, Aidenbach, Germany). After centrifugation at 400 *g* for 5 minutes and resuspension in RPMI cell culture medium, cell numbers were assessed flow cytometrically using a 3L-Cytek Aurora with SpectroFlo software version 3.1.0 (Cytek Biosciences, Fremont, CA, USA). Three micromolars of 4′,6-diamidino-2-phenylindole (BioLegend, San Diego, CA, USA) was used for live and dead differentiation. Subsequently, 5 × 10^5^ live PBMCs each were seeded into two wells of a Human IFN-gamma ELISpot (R&D Systems, Minneapolis, MN, USA) plate. One well was left without any re-stimulation as negative control, and the other one was supplemented with 0.2 µg PepTivator SARS-CoV-2 Prot_S (Miltenyi Biotec, Bergisch-Gladbach, Germany)—a pool of lyophilized peptides, consisting mainly of 15-mer sequences with 11 amino acids overlapping, covering the immunodominant sequence domains of the surface glycoprotein (“S”) of SARS-Coronavirus 2 (GenBank MN908947.3, Protein QHD43416.1). Sequences covered amino acids 304–338, 421–475, 492–519, 683–707, 741–770, 785–802, and 885–1273 (sequence end). Plates were left for 24 hours at 37°C under a 5% CO_2_ atmosphere (Binder, Tuttlingen, Germany). The next day, ELISpot plates were washed four times, IFN-γ detection antibodies were added, and incubation was done overnight at 2°C–8°C. Again, on the next day, plates were washed four times and conjugated AP streptavidin was added to each well. After 2 hours of incubation in the dark at room temperature, plates were washed again four times. Finally, the colored substrate was added, plates were incubated for 30 minutes in the dark at room temperature, and after a final single washing cycle, the plate was dried at 37°C for 30 minutes and scanned. The spot-forming units were counted via ImmunoSpot 5.0 Analyzer with software version 5.0.9.15 (CTL Europe, Bonn, Germany).

### T cell re-stimulation with BNT162b2 and staining for memory marker and intracellular cytokines

PBMCs were thawed and counted via flow cytometry as described in 2.4. For each participant, 8 × 10^5^ live PBMCs were seeded into each of four wells of a 96-well U-bottom tissue culture plate. Two wells remained without any re-stimulation as a negative control. To the other two wells, 1 µg each of BNT162b2 vaccine (Pfizer-BioNTech, Mainz, Germany) was added for re-stimulation. After 20 hours, 1 µg of brefeldin A (Sigma-Aldrich, St. Louis, MO, USA) was added to all wells and left for another 4 hours. Duplicates were pooled, centrifuged at 400 *g* for 5 minutes, and resuspended in autoMACS Running Buffer (Miltenyi Biotec, Bergisch Gladbach, Germany). For live and dead cell differentiation, cells were resuspended in a 1:2,000 dilution of Zombie NIR (BioLegend, San Diego, CA, USA) in PBS and were incubated for 20 minutes in the dark at room temperature. In preparation for antibody staining, non-specific binding sites were blocked using 2.5 µL FCS (Fisher Scientific, Pittsburgh, PA, USA), 1.25 µL TrueStain Monocyte Blocker, and 1.25 µL Human TruStain FcX (BioLegend, San Diego, CA, USA) for 15 minutes on ice. For surface staining, the following antibody:fluorophore combinations were used per sample: 0.125 µg CD3:FITC (clone UCHT1), 0.0156 µg CD4:BV750 (clone SK3), 0.0625 µg CD8:BV570 (clone RPA-T8), 0.125 µg CD25:APC (clone BC96), 0.25 µg CD27:BV605 (clone O323), 0.125 µg CD45RA:APC-Fire750 (clone HI100), 0.125 µg CD45RO:BV421 (clone UCHL1), 1 µg Fas-L:PE (clone NOK1) (BioLegend, San Diego, CA, USA), and 2 µL CD137:PE-Vio615 (clone REA765) (Miltenyi Biotec, Bergisch-Gladbach, Germany), and incubated for 15 minutes in the dark on ice. Samples were then fixed for 20 minutes in the dark at room temperature using fixation buffer (BD Bioscience, Franklin Lakes, NJ, USA). In preparation for intracellular staining, samples were washed three times with intracellular permeabilization wash buffer (BioLegend, San Diego, CA, USA). Again, the non-specific binding sites were blocked as mentioned above. For the subsequent intracellular staining, the following antibody:fluorophore combinations were used per sample: 0.5 µg IFNγ:PerCP-Cy5.5 (clone 4S.B3), 0.125 µg IL-2:BV650 (clone MQ1-17H12), and 1 µg TNFα:PE-Cy7 (clone Mab11) (BioLegend, San Diego, CA, USA). After 30 minutes of incubation in the dark at room temperature, a final wash step and flow cytometric measurements were performed using Cytek Aurora with SpectroFlo software version 3.1.0.

### Statistics

Data were tested for Gaussian distribution using the Kolmogorov-Smirnov test. Pairwise comparisons following Gaussian distribution were performed using the *t*-test for two groups. For data not following Gaussian distribution, the Wilcoxon matched-pairs signed rank was performed for two groups. Unpaired comparisons of three groups were performed via Kruskal-Wallis followed by Dunn’s multiple comparisons tests. Contingency table analyses were performed via χ^2^ test (for gender data). Correlation analyses were performed via Spearman rank correlation. Statistical assays were performed with GraphPad InStat version 3.10 for Windows (GraphPad Software, San Diego, CA, USA) or IBM SPSS Statistics Version 27 (IBM, Armonk, NY, USA). Graphs were created with SigmaPlot 13.0 (Inpixon, Palo Alto, CA, USA). Figure S1 was created with BioRender.com.

## Results

### The different vaccination regimens were equally efficient in preventing subsequent infection

In the present study, we investigated the immune response toward the SARS-CoV-2 spike protein at 18 months after primary immunization. To that extent, we sampled 176 employees of the University Medical Center Rostock. These study participants fell into three groups, defined by the initial vaccination regimen. The first group (*n* = 53) completed their primary immunization with two doses of the adenoviral vector vaccine (from here on termed AZD1222/AZD1222). The second group (*n* = 63) received AZD1222 for their first dose and the mRNA vaccine BNT162b2 for their second (AZD1222/BNT162b2), and the third group (*n* = 60) received two doses of BNT162b2 (BNT162b2/BNT162b2). The third dose at about 9–10 months after first immunization was an mRNA vaccine for all study participants, 12 of whom received mRNA-1273 and all others BNT162b2. By 18 months after primary immunization, very few study participants—one in the AZD1222/AZD1222, two in the AZD1222/BNT162b2, and five in the BNT162b2/BNT162b2 group—had received a fourth dose (Table 1; Fig. S1).

**TABLE 1:** Demographics of study participants

	AZD1222/AZD1222 *n* = 53	AZD1222/BNT162b2 *n* = 63	BNT162b2/BNT162b2 *n* = 60	*P* value
Male (*n*) /female (*n*)	20/33	11/52	17/43	0.049^*a*^
Median age (years) (min–max)	44 (23–66)	43 (22–62)	48 (24–64)	0.101^*b*^
Time from second dose to third immunization (days)	196	203	291	
Third immunization: BNT162b2/mRNA-1273 (*n*/*n*)	47/6	55/4	55/2	
Fourth immunization: BNT162b2/m RNA-1273 (*n*/*n*)	1/0	2/0	5/0	
Infection within 12 months after first immunization (*n*)	3	3	3	
Infection between 12 and 18 months after first imm. (*n*)	28	45	38	0.1178^*a*^
Time of infection (median days after first immunization)	387	396	456	<0.0001^*b*^
Immunized only (no infection) (*n*)	25	18	22	

^*a*^ χ^2^ test.

^*b*^ Kruskal-Wallis Test.

Infections with SARS-CoV-2 occurred in all three vaccination groups. One participant had been infected before first immunization, and another one was positive for anti-nucleocapsid antibodies within the first 6 months after first vaccination yet was unaware of the exact timing. Both belonged to the AZD1222/BNT162b2 group. In total, there were three participants in each group that had been infected within the first 12 months after first immunization. Between 12 and 18 months after first immunization, a total of 28 participants in group 1 (53%), 45 in group 2 (71%), and 38 in group 3 (63%) had recovered from at least one infection. And while the frequencies of infection among the three vaccination groups were comparable, the timing was different. The median time between first immunization and infection was 387 and 396 days post primary infection for the AZD1222/AZD1222 and AZD/BNT162b2 groups, respectively, yet it was 456 days for the BNT162b2/BNT162b2 group, and this difference was statistically significant. We used this information to differentiate between an immune memory to vaccination only and a memory resulting from vaccination plus infection. Table 1 summarizes the demographics of the study population. All participants are part of a longitudinal vaccine study. Antibodies against the nucleocapsid are assessed every 6 months, and the participants are also asked about symptoms of a respiratory infection, including rapid tests performed and PCR test results, in order to monitor their infection status in the long term.

### Primary immunization with BNT162b2 exerted a long-lasting benefit on the humoral response toward the SARS-CoV-2 spike protein

**Fig 1:**
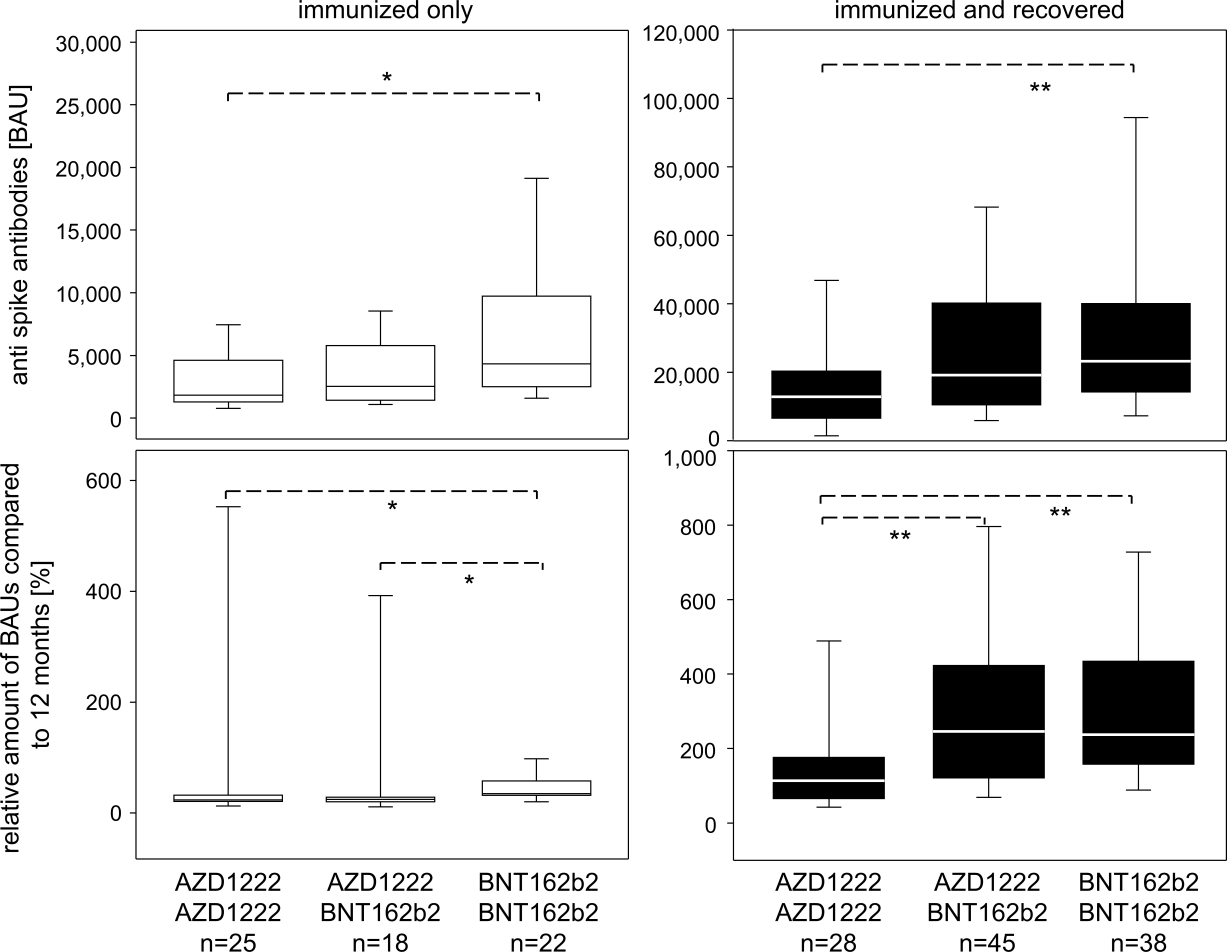
Primary immunization with BNT162b2 exerts long-lasting benefits on the humoral response toward the SARS-CoV-2 spike protein. Upper box plots show anti-SARS-CoV-2 spike RBD antibodies at 18 months after primary immunization as measured in BAU/mL. Study participants who were immunized only are represented on the left, and those who had recovered from at least one infection are shown on the right. Lower box plots show relative amounts of BAUs at 18 months compared with the values measured at 12 months and indicate decreases in the absence of infection (left) and increases after recovery (right). Comparisons between vaccination groups were performed via Kruskal-Wallis tests and resulted in *P* values of 0.0115 (upper left), 0.0066 (upper right), 0.0057 (lower left), and 0.0008 (lower right). Post hoc analyses according to Dunn’s multiple comparison revealed bilateral differences, *P < 0.05 and **P < 0.01.

We were interested whether differences in vector- and mRNA vaccine-induced immune responses observed at 6 and 12 months after primary immunization would persist for longer. To that extent, we focused on the study participants that had not yet been infected by SARS-CoV-2 and quantified at 18 months both, the amount of serum IgG antibodies against RBD of the spike protein and their neutralization capacities. We observed that the AZD1222/AZD1222 and AZD1222/BNT162b2 groups presented with medians of 1,598 and 2,482 BAU/mL, compared with a median of 4,323 for the BNT162b2/BNT162b2 group (Fig. 1, upper left panel). A comparison between these three groups via the Kruskal-Wallis test resulted in a *P* value of 0.0115 and indicated a significant difference between the AZD1222/AZD1222 and BNT162b2/BNT162b2 groups. A comparison to the anti-spike antibodies measured at 12 months after primary immunization revealed a reduction to 22.5% for AZD1222/AZD1222, to 20.0% for AZD1222/BNT162b2, and to 33.5% for the BNT162b2/BNT162b2 group. A comparison between these three groups via the Kruskal-Wallis test resulted in a *P* value of 0.0057 and indicated a significantly smaller loss for the BNT162b2/BNT162b2 group (Fig. 1, left panels). The positive correlation between anti-spike antibodies measured in BAU/mL and the corresponding neutralization capacities shown in Fig. S2 confirmed higher titers coupled to higher functionality. In summary, homologous primary immunization with the mRNA vaccine BNT162b2 not only induced higher amounts of serum anti-spike antibodies and led to an attenuated decline over time but also suggested an elevated neutralization capacity 18 months after the first immunization.

To investigate the impact of SARS-Cov-2 infection on the longevity of the immune response, we focused on those study participants who in addition to primary and subsequent immunizations had recovered from at least one SARS-CoV-2 infection. We observed that the AZD1222/AZD1222 and AZD1222/BNT162b2 groups presented with medians of 13,134 and 19,111 BAU/mL, compared with a median of 23,130 for the BNT162b2/BNT162b2 group. A comparison between these three groups via the Kruskal-Wallis test resulted in a *P* value of 0.0259 and indicated significantly higher serum antibody concentrations for the BNT162b2/BNT162b2 compared with the AZD1222/AZD1222 group. A comparison to the anti-spike antibodies measured at 12 months after primary immunization revealed an increase to 114% for AZD1222/AZD1222, to 245% for AZD1222/BNT162b2, and to 224% for the BNT162b2/BNT162b2 groups. A between-groups comparison via the Kruskal-Wallis test resulted in a *P* value of 0.0031 and indicated a significantly smaller increase for the homologously primed AZD1222/AZD1222 group (Fig. 1, right panels). In summary, having been primed with the mRNA vaccine BNT162b2 facilitated enhanced humoral immune response to infection with the virus at later time points.

### Primary immunization with BNT162b2 promoted benefits for the cellular response toward the SARS-CoV-2 spike protein

**Fig 2:**
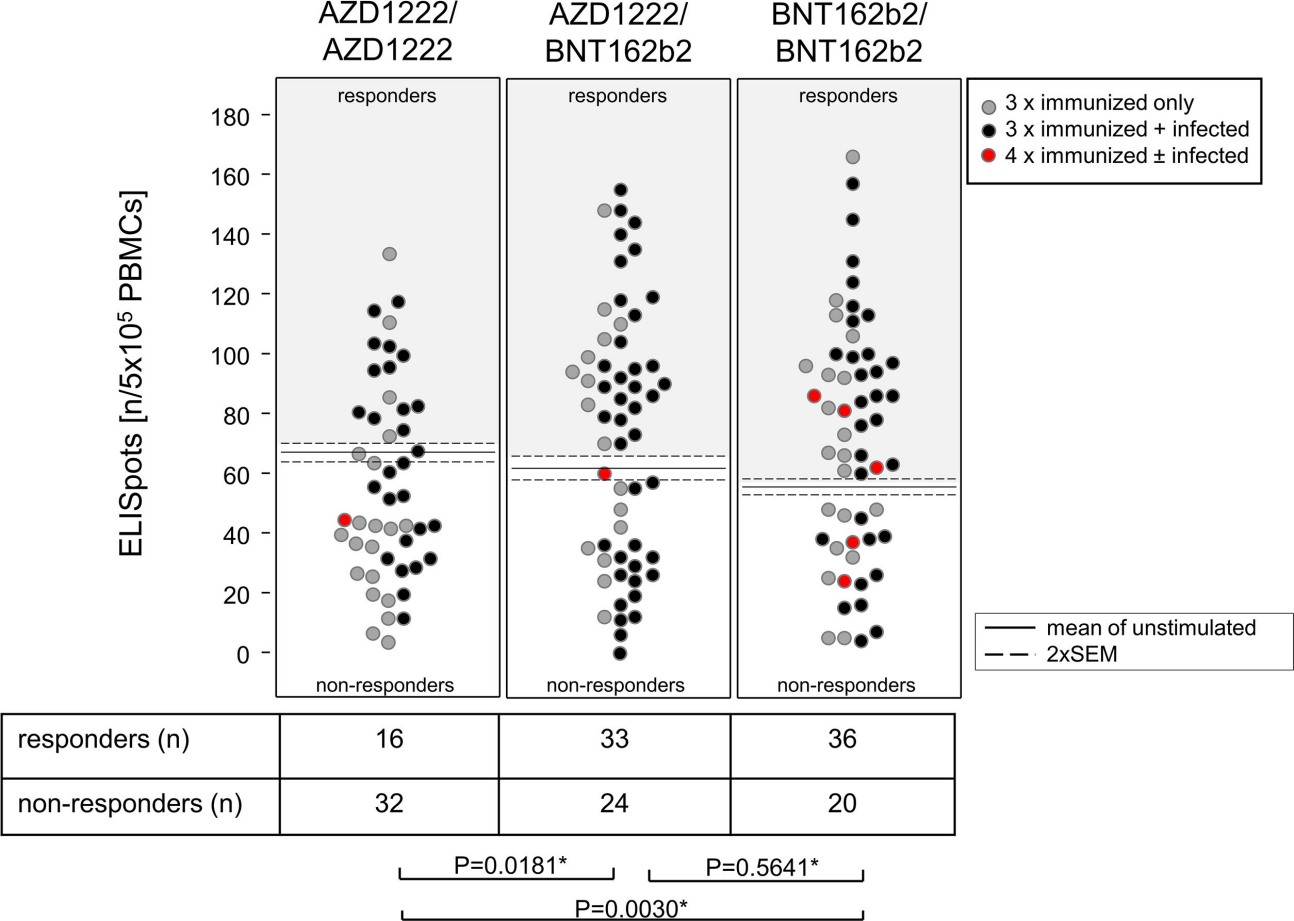
Primary immunization with BNT162b2 promoted benefits for the cellular response toward the SARS-CoV-2 spike protein. Dot plots show absolute numbers of IFNg-producing ELISpots resulting from 5 × 10^5 PBMCs stimulated with peptide pools representing the spike protein. Horizontal lines indicate means ± 2 SEM of IFNg-producing ELISpots present in the absence of peptide re-stimulation. Each dot represents one individual, and in case of more than mean + 2 SEM IFNg-producing ELISpots, individuals were considered responders. The table gives numbers of responders and non-responders resulting from each vaccination regimen. Fisher´s exact tests yield a significantly lower frequency of responders for the AZD1222/AZD1222 group.

In order to investigate whether the various vaccination regimen also impacted on the pro-inflammatory T cell memory, we performed ELISpot assays and measured the IFNγ release in response to a wild-type Wuhan-Hu-1-specific peptide pool. Figure 2 presents the absolute numbers of spot-forming cells derived from 500.000 PBMC each. The cut-off above which study participants were considered responders was the mean (+2 SEM) of spot-forming cells observed in the absence of any re-stimulation. Our results indicated that both homologous and heterologous vaccination regimens with BNT162b2 elicited significantly higher frequencies of responders to non-responders compared with the AZD1222/AZD1222 group. However, as opposed to the antibody response, prior infection did not result in increased numbers of IFNγ producers and that held true for all vaccination groups. Likewise, even though the numbers are very low, a fourth immunization did not seem to result in increased IFNγ responses.

### All three vaccination regimens gave rise to comparable T cell memories

**Fig 3:**
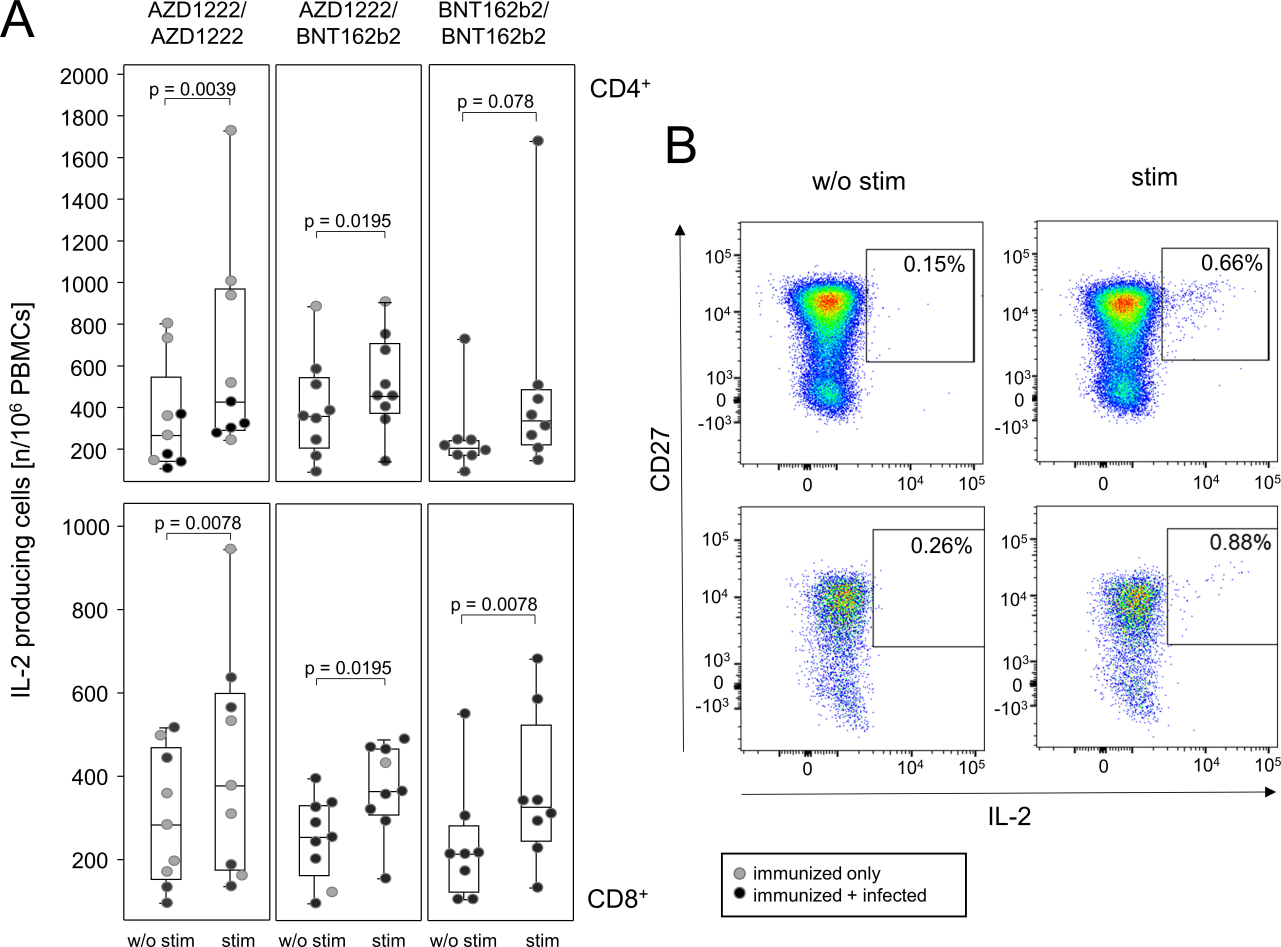
All three vaccination regimens give rise to comparable T cell memories. Combinations of dot and box plots show the numbers of IL-2 producing CD4^+^ T helper (A, upper panel) and CD8^+^ cytotoxic T cells (A, lower panel) after *in vitro* re-stimulation of 10^6^ live PBMCs. Each dot represents one participant. Exemplary plots for CD4^+^ (upper panels) and CD8^+^ (lower panels) CD45RO^+^/CD45RA^−^ T cells expressing CD27 and IL-2 in the absence and after re-stimulation are shown on the right. Comparisons were performed using Wilcoxon matched-pairs signed rank tests; exact *P* values are reported.

To gain a deeper insight into the T cell memory, peripheral PBMCs were re-stimulated *in vitro* using the BNT162b2 vaccine and were then subjected to intracellular cytokine staining. Independent of the primary immunization regimen, the individuals tested—eight or nine per vaccination group—collectively showed an increased IL-2 production after re-stimulation and that held true for the CD4 as well as the CD8 compartments (Fig. 3, left hand panels). We cannot rule out though that innate stimuli resulting from the mRNA vaccine had impact on our readout. Upon closer inspection, IL-2 production was predominantly confined to CD45RO^+^/CD45RA^−^ memory cells (Fig. 3, right hand panels). A gating scheme is provided in Fig. S3. Note that the numbers analyzed were too low to allow for a discrimination between study participants who were immunized only or who in addition had recovered from infection. Unfortunately, none of the other cytokines tested yielded significant increases following *in vitro* re-stimulation (see Table S1 and S2). In summary, our data suggest that the overall T cell memory measured via IL-2 production was comparable between all three vaccination groups.

## DISCUSSION

Our results show that even at 18 months after primary immunization, study participants who had initially been vaccinated with BNT162b2—either alone or heterologously in combination with AZD1222—still had benefits in terms of the amount of serum antibodies and cellular memory. These findings have two implications: firstly, as infection rates were comparable among the three vaccination regimens, reduced humoral and cellular memory in the AZD1222/AZD1222 group did not lead to a heightened risk of infection. Instead, comparable vaccination efficiencies imply an overabundance of memory in the BNT162b2/BNT162b2 and BNT162b2/AZD1222 groups. However, it remains a possibility that as antibody titers wane with time, the risk of infection may rise sooner in the AZD1222/AZD1222 group, and indeed, infection in the BNT162b2/BNT162b2 group occurred later with respect to the date of primary immunization. It will be important to follow-up over longer time periods if this trend continues in order to define vaccination intervals.

The second implication refers to a different imprinting through either mRNA- or vector-based vaccine. The concept of original antigenic sin implies that the immune system will rely on the first cohort of B cells engaged by an antigen and, upon repeated challenge, will prevent *de novo* responses (19, 20). Two different mechanisms have been debated and suggest that original antigenic sin either relies on active suppression of newly recruited B cell clones into germinal center reactions or is a phenomenon at the serum level (21). An elegant study in mice recently pondered on the latter. In case of zero antigenic distance—as is the case in repeated immunizations with an identical antigen—newly emerging B cells would be outcompeted by antigen experienced ones and would either not have sufficient affinity to exit the germinal center as plasma cell or would secrete antibodies that are hardly detectable (22). However, our results favor suppression. If affinities were responsible for the reduced humoral response resulting from the AZD1222/AZD1222 priming, subsequent immunizations with BNT162b2 should foster higher affinity B, memory, and plasma cells and allow the AZD1222 primed study participants to catch up. This does not seem to happen as shown by our results at 18 months after primary immunization.

Interestingly, whether T cell responses are subject to original antigenic sin is not as clear cut. While the ELISpot shows a reduced cellular memory for the AZD1222/AZD1222 group, heterologous priming with AZD1222 and BNT162b2 seems to accelerate the cellular memory to the level of BNT162b2/BNT162b2 primed individuals. Of note, the T cell memory assessed via intracellular IL-2 production appears comparable for all three vaccination groups; however, the numbers of analyzed individuals were much lower here and may therefore not be representative for the whole cohort.

Our results will have consequences for future vaccine designs. Not only does the mRNA vaccine elicit stronger T and B cell responses from the start (9, 13, 16, 23–25) but also antibody titers persist for longer (26) and may therefore provide better protection (27, 28). Another aspect to consider when designing vaccines is the ease and speed required to modify vaccines and adapt to variants of concern. Against the backdrop of original antigenic sin, repeated immunizations should be performed with antigens that are as distant from the original vaccine as possible (22).

There are limitations to our study: first of all, we here investigated solely Caucasians and can therefore only speculate if comparable results will apply to other ethnicities. Second, we did not have record on comorbidities beyond SARS-CoV-2 infection and cannot exclude that factors we did not monitor had impact on our findings. Third, we have no documentation on the variants of concern the study participants were infected with; however, based on the timing between January 2022 and June 2022 and complemented by public sequencing data, we assume they were BA.1 or BA.2, respectively (https://ourworldindata.org/grapher/covid-variants-bar?time=2022-06-06). We therefore cannot conclusively resolve if vaccine efficiency differs depending on the variant of concern. It will be interesting to follow-up how protein-based vaccines like Nuvaxovid will compare and if any of the vaccines is more efficient than another in preventing the lasting sequelae of infection, long COVID.

## Data Availability

The authors agree that any materials and data that are reasonably requested by members of the scientific community will be made available in a timely fashion for non-commercial purposes.

## SUPPLEMENTAL MATERIAL

Fig. S1 jvi.01912-23-s0001.pdf Study design.

Fig. S2 jvi.01912-23-s0002.pdf Correlation between BAU and neutralizing capacity.

Fig. S3 jvi.01912-23-s0003.pdf Gating strategy.

Tables S1 and S2 jvi.01912-23-s0004.pdf T cell cytokine responses.

ASM does not own the copyrights to Supplemental Material that may be linked to, or accessed through, an article. The authors have granted ASM a non-exclusive, world-wide license to publish the Supplemental Material files. Please contact the corresponding author directly for reuse.
