# Supplementary material for: The varying extent of humoral and cellular immune responses to either vector- or RNA-based SARS-CoV-2 vaccines persists for at least 18 months and is independent of infection
Source: J Virol. 2024 Mar 19;98(4):e01912-23. doi: 10.1128/jvi.01912-23 (PMC11019912; doi:10.1128/jvi.01912-23)
Supplement: Fig. S1 — Study design. [file jvi.01912-23-s0001.pdf]

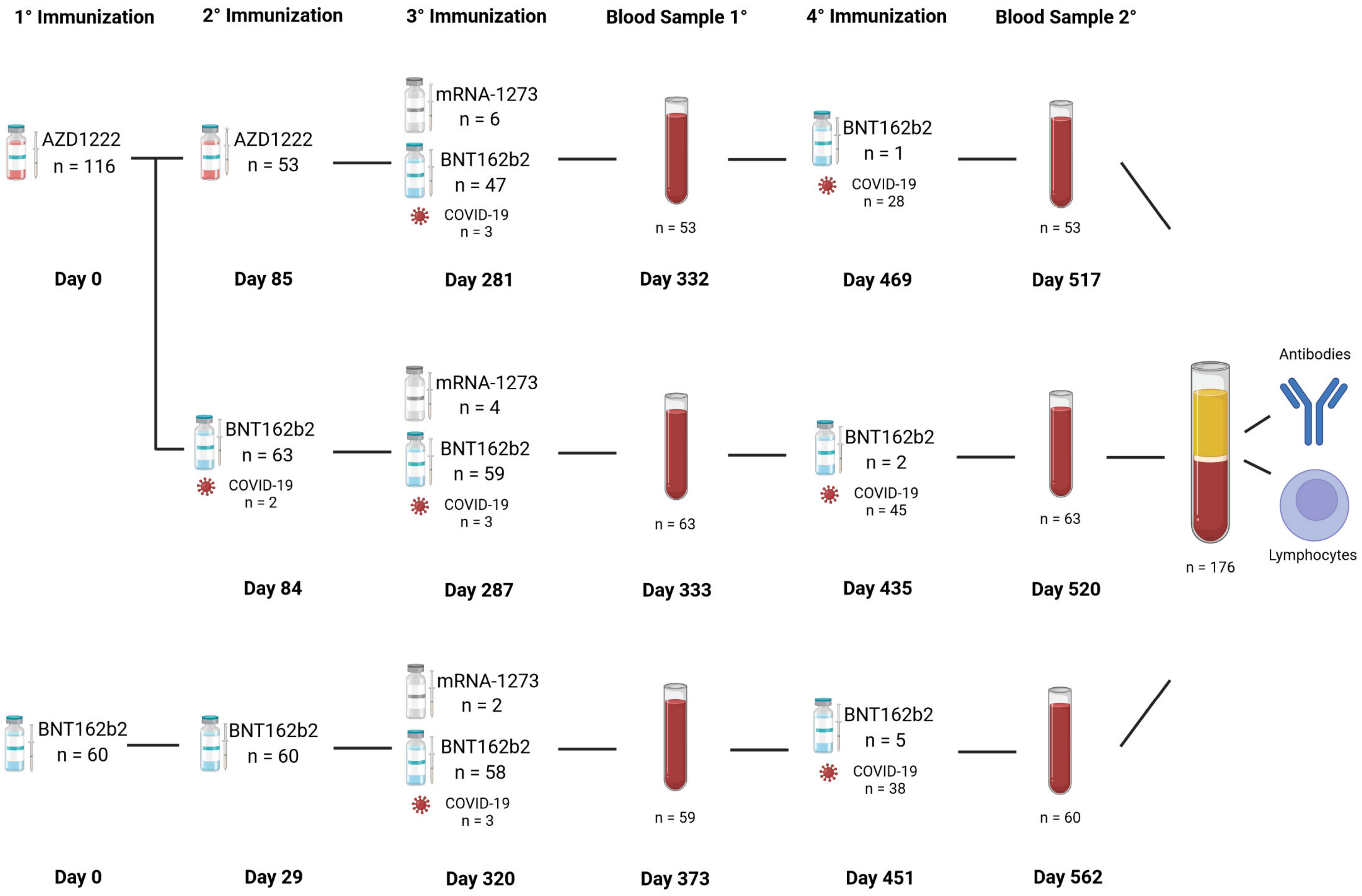

Supplemental Figure 1

Supplemental Figure 1 Study Design.

Shown is the composition of the different vaccination groups for all 176 study participants depending on their primary immunization, which is also used here consecutively to distinguish vaccination groups. Homologous primary immunization with "AZD1222/AZD1222" (n = 53) or "BNT162b/BNT162b2" (n = 60) as well as heterologous "AZD1222/BNT162b2" (n = 63) represent the respective vaccination groups regardless of subsequent immunizations; AZD1222 = ChAdOx1 from AstraZeneca; BNT162b2 from Pfizer-BioNTech; mRNA-1273 from Moderna. First doses of BNT162b2 were obtained between Dec 27th and 31st, 2020, first doses of AZD1222 between middle and end of February 2021. The sequence of immunizations, identified COVID-19 diseases and also the time points of blood sampling are displayed.
