# Supplementary material for: The varying extent of humoral and cellular immune responses to either vector- or RNA-based SARS-CoV-2 vaccines persists for at least 18 months and is independent of infection
Source: J Virol. 2024 Mar 19;98(4):e01912-23. doi: 10.1128/jvi.01912-23 (PMC11019912; doi:10.1128/jvi.01912-23)
Supplement: Fig. S2 — Correlation between BAU and neutralizing capacity. [file jvi.01912-23-s0002.pdf]

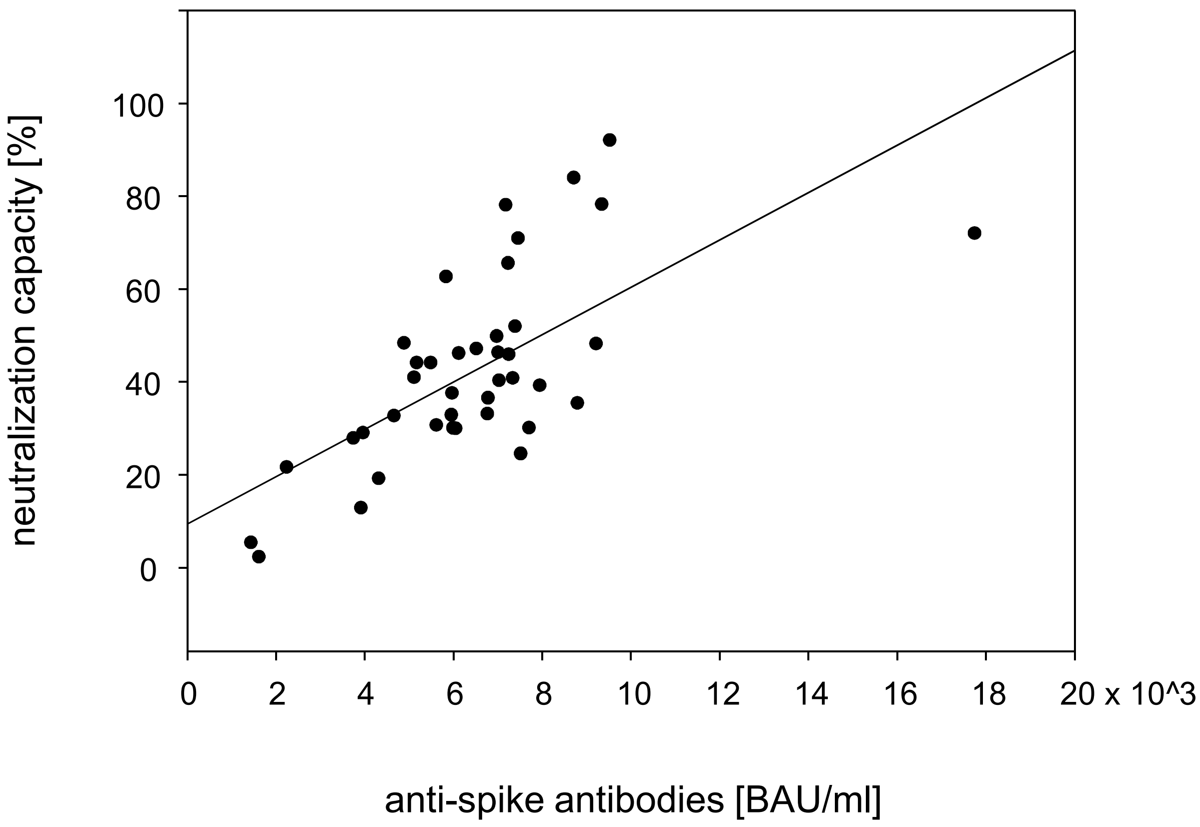

Supplemental Figure 2

Supplemental Figure 2 Correlation between BAU/mL and neutralizing capacity.

Linear regression shows a statistically significant correlation between the amount of antibodies measured in BAU/ml and the neutralization capacity in %. Spearman rank correlation for 40 serum samples diluted at 1:200 resulted in $r = 0.6394$; 95% CI = 0.4014 to 0.7965; and a two tailed p-value < 0.0001.
