# Supplementary material for: The varying extent of humoral and cellular immune responses to either vector- or RNA-based SARS-CoV-2 vaccines persists for at least 18 months and is independent of infection
Source: J Virol. 2024 Mar 19;98(4):e01912-23. doi: 10.1128/jvi.01912-23 (PMC11019912; doi:10.1128/jvi.01912-23)
Supplement: Fig. S3 — Gating strategy. [file jvi.01912-23-s0003.pdf]

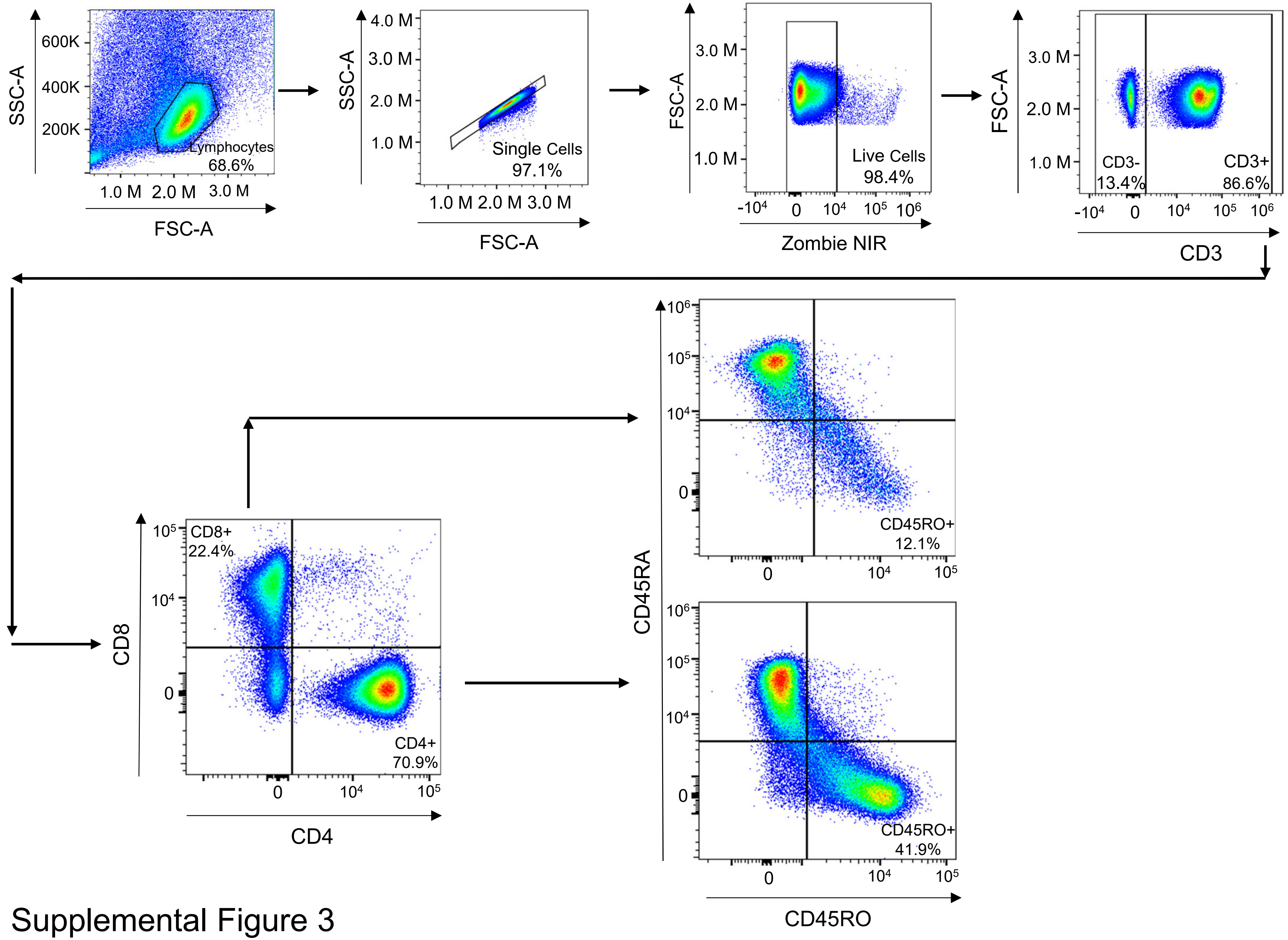

Supplemental Figure 3

Supplemental Figure 3 Gating strategy for the analyses of intracellular T cell cytokine production.

Flow plots exemplify the strategy employed for gating on $CD4^+$ and $CD8^+$ memory T cells for subsequent intracellular cytokine analysis. After identification of single cells, dead cells were excluded, $CD3^+$ T cells gated and then $CD8^+$ cytotoxic and $CD4^+$ T helper cells differentiated. These were further characterized according to CD45RO and CD45RA. Memory T cells were defined as $CD45RO^+$ and $CD45RA^-$ cells.
