# Supplementary material for: The varying extent of humoral and cellular immune responses to either vector- or RNA-based SARS-CoV-2 vaccines persists for at least 18 months and is independent of infection
Source: J Virol. 2024 Mar 19;98(4):e01912-23. doi: 10.1128/jvi.01912-23 (PMC11019912; doi:10.1128/jvi.01912-23)
Supplement: Tables S1 and S2 — T cell cytokine responses. [file jvi.01912-23-s0004.pdf]

Supplemental Table 1. In vitro restimulation of PBMC with BNT162b2 yielded a strong increase in IL-2 producing CD4⁺ memory cells

	AZD1222/AZD1222 positive cells/10^6 PBMC [n]			AZD1222/BNT162b2 positive cells/10^6 PBMC [n]			BNT162b2/BNT162b2 positive cells/10^6 PBMC [n]		
	non-stimulated	stimulated	p-value*	non-stimulated	stimulated	p-value*	non-stimulated	stimulated	p-value*
**$CD4^+$ T helper cells**									
IFN-γ	2948	3151	0.953	2872	2922	0.26	2970	3446	0.017
TNFα	185	217	0.139	186	234	0.260	199	224	0.263
Fas-L	2467	1976	0.051	2281	2012	0.086	2399	2230	0.05
CD137	2577	1989	0.038	2820	2631	0.214	2971	2963	0.779
CD25	20998	20900	0.859	24086	24461	0.260	22522	23280	0.123
CD45RO+ CD45RA+ CD27+ IL-2+	54	117	0.374	55	74	0.066	113	209	0.036
CD45RO+ CD45RA+ CD27- IL-2+	28	59	0.018	28	38	0.400	13	28	0.046
CD45RO+ CD45RA- CD27+ IL-2+	777	1350	0.008	728	1300	0.008	604	1206	0.012
CD45RO+ CD45RA- CD27- IL-2+	166	247	0.038	96	178	0.008	45	75	0.042

*Wilcoxon matched pairs signed rank test

Supplemental Table 2 In vitro restimulation of PBMC with BNT162b2 yielded a strong increase in IL-2 producing CD8⁺ memory cells

	AZD1222/AZD1222			AZD1222/BNT162b2			BNT162b2/BNT162b2		
	positive cells/10^6 PBMC [n]			positive cells/10^6 PBMC [n]			positive cells/10^6 PBMC [n]		
	non-stimulated	stimulated	p-value*	non-stimulated	stimulated	p-value*	non-stimulated	stimulated	p-value*
**$CD8^+$ T-cells**									
IFN-γ	871	915	0.594	838	1023	0.110	994	1023	0.484
TNFα	135	224	0.066	140	258	0.028	152	167	0.889
Fas-L	748	666	0.051	801	782	0.859	972	861	0.889
CD137	1025	785	0.161	928	1045	0.155	954	960	0.214
CD25	3422	3635	0.401	1603	2065	0.011	1698	1810	0.401
CD45RO+ CD45RA+ CD27+ IL-2+	96	156	0.263	112	164	0.139	89	135	0.012
CD45RO+ CD45RA+ CD27- IL-2+	43	87	0.028	53	85	0.069	108	222	0.012
CD45RO+ CD45RA- CD27+ IL-2+	75	136	0.021	64	108	0.086	53	95	0.093
CD45RO+ CD45RA- CD27- IL-2+	21	41	0.326	14	20	0.123	17	34	0.049

*Wilcoxon matched pairs signed rank test
